# Understanding the impact of sex and stage differences on melanoma cancer patient survival: a SEER-based study

**DOI:** 10.1038/s41416-020-01144-5

**Published:** 2020-11-04

**Authors:** Aiden J. Smith, Paul C. Lambert, Mark J. Rutherford

**Affiliations:** 1 Department of Health Sciences, University of Leicester, Leicester, UK; 2 Department of Medical Epidemiology and Biostatistics, Karolinska Institutet, Stockholm, Sweden

**Keywords:** Cancer epidemiology, Cancer, Melanoma

## Abstract

**Background:**

This paper investigates the difference in survival of melanoma patients across stage and sex by utilising net survival measures. Metrics are presented at both the individual and population level.

**Methods:**

Flexible parametric models were fitted to estimate life-expectancy metrics to be applied to a group of 104,938 subjects with a melanoma skin cancer diagnosis from 2000 to 2017. Period analysis was used for better predictions for newly diagnosed patients, and missing-stage information was imputed for 9918 patients. Female relative survival was assigned to male subjects to demonstrate the survival discrepancies experienced between sexes.

**Results:**

At the age of 60, males diagnosed at the regional stage lose an average of 4.99 years of life compared to the general population, and females lose 4.79 years, demonstrating the sex variation in expected mortality. In 2017, males contributed 3545 more life years lost than females, and a potential 1931 life years could be preserved if sex differences in survival were eliminated.

**Conclusions:**

This study demonstrates the survival differences across population subgroups as a result of a melanoma cancer diagnosis. Females experience better prognosis across age and stage at diagnosis; however, further investigation is necessary to better understand the mechanisms behind this difference.

## Background

There have been worldwide increases in melanoma skin cancer incidence over the last several decades,^1–4^ coupled with steady improvement in patient survival for diagnosed individuals.^5^ In spite of the progress seen over time, variation in survival across population subgroups still persists.^6^ Fair comparisons of cancer patient survival can be made across population subgroups (such as those defined by sex, geographical location, socioeconomic status, calendar periods of diagnosis and age) by accounting for differential background (other-cause) mortality rates, and reporting net survival measures that are solely impacted by the extra mortality associated with a cancer diagnosis.^7,8^

Disparities in cancer survival between sexes across cancer sites are well documented with females often experiencing better prognosis,^9^ including for melanoma.^10^ At a population level, it is of interest to understand the magnitude of this difference and, were this disparity to be eliminated, the amount of life years that could be preserved. Standard net survival metrics (e.g. 1- and 5-year age-standardised relative survival) are often difficult to interpret and are not tangible metrics to quantify the overall impact of cancer, making it difficult to provide meaningful statistics that are useful for decision makers.

An alternative metric that has become increasingly popular is the average life expectancy. From the same statistical model in the relative survival framework, it is possible, under a range of assumptions that have been shown to be plausible for adult cancer patient populations,^11^ to estimate 1- and 5-year stage-specific relative survival, as well as loss in life expectancy (LEL) for each stage at diagnosis relative to a disease-free member of the general population.^12^ These measures are intuitive in that they can be manipulated to explain the loss in life expectancy of individuals across their whole lifespan post diagnosis,^13,14^ as well as quantify the burden of a specific disease on the population as a whole. These population measures can be used to inform policy makers in regard to the population benefit of potential investments into screening or treatment programmes.

Various other metrics can be derived from the measures of life expectancy providing a range of reporting options that show different aspects of cancer prognosis based on an individual’s characteristics. The proportion of expected life lost (PELL) can be calculated to make LEL more comparable across the age spectrum, given the premise that older patients will naturally have less- available life years to lose. Total life years lost (TLYL) can be calculated to understand the differing impact of each stage at diagnosis across the population.

This study utilises US SEER data to estimate the impact of melanoma skin cancer at both the individual and population levels, across stages at diagnosis using an array of metrics. Population metrics were constructed to demonstrate the societal burden of melanoma and determine the number of life years, which could be preserved if both sexes experienced equivalent stage-specific relative survival.

## Methods

### Data resources

Individual case listings obtained for all individuals diagnosed with a melanoma skin cancer diagnosis between 2000 and 2017 were extracted from the Surveillance, Epidemiology, and End Results Program (SEER) (9 Registries) database from the United States National Cancer Institute SEER*Stat tool,^15^ and analysed in Stata. Alongside staging information, individual patient characteristic data, such as age at diagnosis are collected and provide the ability to assess survival differences between population subgroups. Melanoma cases were extracted from the SEER database using the primary tumour site ICD-O-3 codes (C44.0–C44.9). Categories for stage at diagnosis are coded in accordance with the SEER summary staging system,^16^ where malignant tumours are defined as localised (invasive tumours confined to the superficial skin), regional (tumours that have invaded the deeper skin tissues, organs and lymphatic system surrounding the primary tumour site) and distant (tumours that have metastasised beyond the region surrounding the primary tumour site into the rest of the body). Tumours coded as in situ were excluded from the analysis. For patients registered with multiple tumours, only the first tumour was considered.

### Statistical methods

Flexible parametric survival models were fitted using restricted cubic splines to more accurately capture the baseline excess hazard.^17,18^ The models were fitted using the -***stpm2-*** command in Stata*.*^19^ Separate models were fitted for males and females. These models enable the estimation of relative survival, life expectancy, loss in expectation of life and proportion of expected life lost for each of the stages of cancer diagnosis. Expected mortality rates in the general population were incorporated using SEER*Stat US Mortality data for the years 2000–2017, stratified by age, sex, year and race.^15^

The fitted models included age and stage at diagnosis. Age was modelled using splines to allow for non-linearity and modelled as a continuous variable. Relative survival estimates were Winsorized based on age at diagnosis to aid with model stability.^20^ Both age and stage at diagnosis were assumed to have time-dependent effects. Multiple imputation was performed on 9918 missing observations (9.45% of data) in the stage at diagnosis variable.^21^ The imputation models were fitted separately for sex, and also included age at diagnosis, year of diagnosis, anatomical tumour sub-site and tumour grade (stage missingness distributions of these covariates are presented in Supplementary Table 1). Missing-stage data were imputed using 30 imputation iterations,^22^ and analysis was conducted using each imputed dataset and combined using Rubin’s rules to obtain summary estimates and confidence intervals of the metrics (see Supplementary Material: Statistical Methods Appendix for further details).

A period analysis with a period window of 5 years between 2013 and 2017 was used as it allows for better predictions of prognosis for newly diagnosed patients compared to traditional cohort approaches.^23,24^ Period analysis involves only using follow-up experience that falls within the period window. In doing so, we use the short-term survival experience from those diagnosed more recently (diagnosed in 2013–2017), and the long-term survival experience is contributed from those diagnosed further back in time (back to diagnoses from 2000), whilst the short-term experience of those diagnosed historically is excluded. Period analysis, therefore, provides more accurate, up-to- date predictions for long-term survival, which are not unduly influenced by patients in the cohort who were diagnosed further in the past who generally experience worse survival than more recent diagnoses. LEL and PELL estimates are calculated for each age at diagnosis based on the approach of Andersson et al.^25^ Finally, the total life years lost was calculated for melanoma cases diagnosed in 2017 to demonstrate the societal impact these cancer cases have at a population level, and how the magnitude of this impact varies by stage at diagnosis. Furthermore, by assigning the relative survival of females to males, it is possible to calculate the population-level life years lost, which is a result of the disparity in sex-specific relative survival.

All statistical analyses were conducted using Stata 16.^26^

## Results

In total, 133,690 patients were extracted from the SEER database with a melanoma cancer code. After data cleaning, 104,938 patients with a melanoma skin cancer diagnosis were included in the analysis. Patients were excluded if they did not have complete date information (n = 28,752). Table 1 shows the number of patients by sex, and the distribution of stage and age at diagnosis. Those diagnosed at a localised stage are younger on average, with those diagnosed at distant stage having the highest mean age at diagnosis. Females are typically diagnosed at an earlier age than males on average in the localised and regional stages; however, they are older on average when diagnosed at the distant stage when compared to males. There are more male cases across all 3 stages; however, there is a larger disparity between sexes in the patients with missing-stage information, with stage information missing from ~3000 more males compared to females, representing a 2.98% increase in the proportion of missing data in the stage distribution. The stage distributions between sexes are markedly different, with a larger proportion of female cases being diagnosed at the localised stage (81.29–75.39%), whereas a higher proportion of both regional and distant cases are found in males. Greater differences are noticeable in the age distributions between the sexes, with a far higher proportion of males being diagnosed over 60 compared to females. A large disparity is noticeable in the under-45 age group, where there is a markedly higher proportion of the female cases when compared to the males (25.34–15.42%).

**Table 1:** Baseline characteristics of melanoma skin cancer patients extracted from the SEER database 2000–2017.

	Male	Female	Total
*N* (Total %)			
	60,351 (57.51)	44,587 (42.49)	104,938 (100)
Stage (sex distribution %)			
Localised	45,499 (75.39)	36,243 (81.29)	81,742 (77.90)
Regional	5993 (9.93)	3731 (8.37)	9724 (9.27)
Distant	2390 (3.96)	1164 (2.61)	3554 (3.39)
Missing	6469 (10.72)	3449 (7.74)	9918 (9.45)
Age group at diagnosis (sex distribution %)			
<45	9307 (15.42)	11,300 (25.34)	20,607 (19.64)
45–60	15,924 (26.39)	12,996 (29.15)	28,920 (27.56)
60–75	21,731 (36.01)	12,238 (27.45)	33,969 (32.37)
>75	13.389 (22.19)	8053 (18.06)	21,442 (20.43)
Mean age at diagnosis (SD)			
Localised	62.23 (15.28)	56.14 (17.22)	59.53 (16.45)
Regional	61.96 (16.91)	59.79 (18.95)	61.13 (17.75)
Distant	63.67 (15.04)	64.49 (16.08)	63.94 (15.39)

**Fig. 1:**
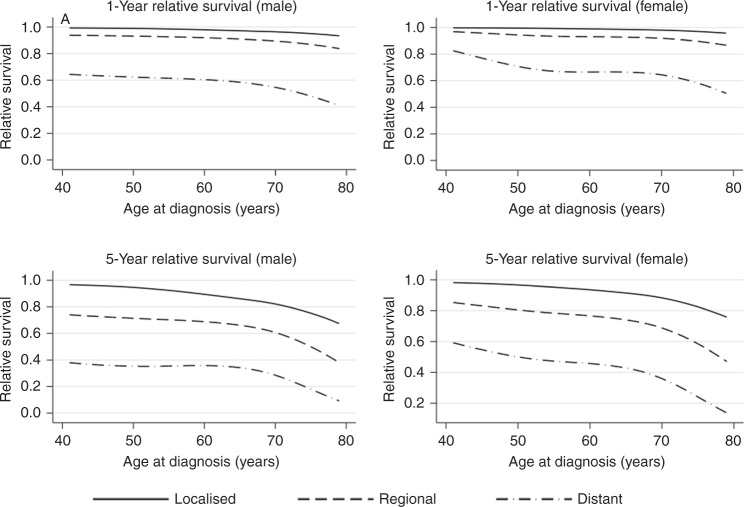
Stage-specific 1- and 5-year relative survival curves as a function of age at diagnosis. Top-left: Male 1-Year relative survival; top-right: Female 1-Year relative survival; bottom-left: Male 5-Year relative survival; bottom-right: Female 5-Year relative survival.

Figure 1 shows the stage- and age-specific 1- and 5-year relative survival, with the worst prognosis estimated for older patients. A noticeable decline occurs around age 70 in both the 1-year and 5-year estimates, although with greater severity in the longer term. There are also marked differences between the estimates for the regional and distant-stage diagnosed patients, with sex differences in survival also being noticeable for all stages of diagnosis.

**Fig. 2:**
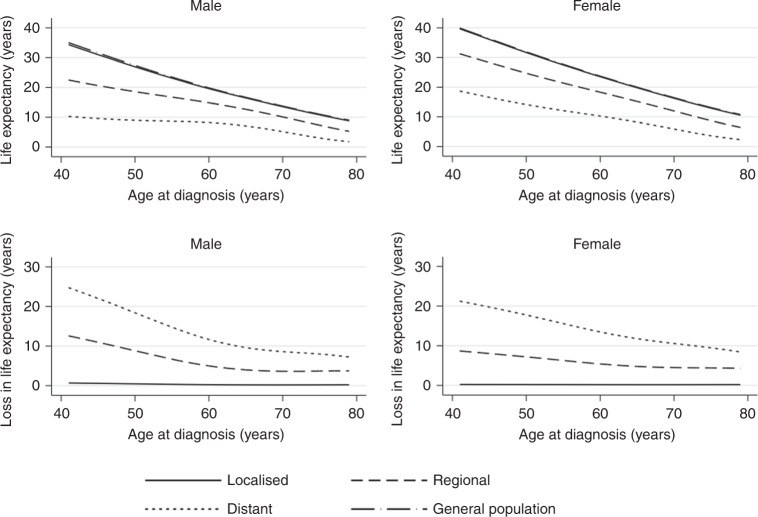
Comparison of life expectancy between stage of diagnosis in cancer patients and the general population across age at diagnosis and sex. Loss in expectation of life in comparison to general population covariate-matched subject stratified by sex and stage at diagnosis across a range of age at diagnosis.

Figure 2 and Table 2 show the life expectancy and loss in expectation of life as a result of a melanoma diagnosis. From the life-expectancy plots, we see that across all ages of diagnosis within the regional and distant stages of diagnosis, there is a reduction in expectation of life compared to that of the general population average. In the localised-stage group, there is very little difference between the general population and those diagnosed with melanoma. Consistent with the 1- and 5-year relative survival estimates, the distant-stage cases have a notably worse prognosis in terms of both the overall life expectancy and the years of expected life lost. A 60-year-old male individual diagnosed with localised melanoma can expect to lose on average 0.25 years of life compared to a similar individual in the general population. This figure increases to 4.99 and 11.73 years, respectively, for regional and distant stage at diagnosis. Comparatively, an 80-year-old male would expect to lose on average 0.24, 3.72 and 6.99 years of life for each given stage, respectively. At the distant stage, a 60-year-old man is expected to lose 59.07% of their remaining expected life and the 80-year-old man to lose 81.74%. When considering sex variation, females have a lower LEL compared to men. Male cases have in general a higher proportion of expected life lost, although this is not consistent across all ages and stages, suggesting that females are impacted either equally or marginally less by a cancer diagnosis when compared to males.

**Table 2:** Age- and stage-specific estimates of life expectancy, loss in life expectancy and proportion of expected life.

	Localised				Regional				Distant				General population LE	
	LE (95% CI)		LEL (PELL %)		LE (95% CI)		LEL (PELL %)		LE (95% CI)		LEL (PELL %)			
Age	Male	Female	Male	Female	Male	Female	Male	Female	Male	Female	Male	Female	Male	Female
40	35.22 (34.88–35.56)	40.69 (40.86–40.51)	0.70 (1.94)	0.17 (0.42)	22.96 (21.21–24.71)	32.72 (34.83–30.62)	12.96 (36.07)	8.14 (19.92)	10.29 (7.49–13.09)	19.89 (14.59–25.20)	25.62 (71.35)	20.97 (51.32)	35.92	40.86
50	26.93 (26.73–27.13)	31.74 (31.60–31.86)	0.46 (1.68)	0.16 (0.50)	18.60 (17.40–19.82)	25.43 (23.78–27.08)	8.78 (32.07)	6.47 (20.29)	8.87 (6.89–10.84)	15.02 (11.34–18.71)	18.52 (67.63)	16.88 (52.91)	27.39	31.90
60	19.70 (19.52–19.70)	23.60 (23.51–23.70)	0.25 (1.26)	0.13 (0.56)	14.87 (14.13–15.60)	18.94 (17.91–19.98)	4.99 (25.16)	4.79 (20.19)	8.13 (6.96–9.30)	11.12 (8.84–13.39)	11.73 (59.07)	12.62 (53.17)	19.86	23.74
70	13.56 (13.49–13.62)	16.33 (16.24–16.43)	0.19 (1.36)	0.13 (0.80)	10.11 (9.55–10.66)	12.31 (11.43–13.18)	3.64 (26.47)	4.15 (25.26)	5.12 (4.35–5.89)	6.28 (4.84–7.71)	8.62 (62.74)	10.19 (61.88)	13.74	16.47
80	8.31 (8.22–8.41)	10.02 (9.88–10.16)	0.24 (2.78)	0.18 (1.85)	4.83 (4.49–5.17)	5.80 (5.25–6.35)	3.72 (43.48)	4.40 (43.16)	1.56 (1.32–1.80)	1.99 (1.66–2.33)	6.99 (81.74)	8.21 (80.45)	8.55	10.20

**Fig. 3:**
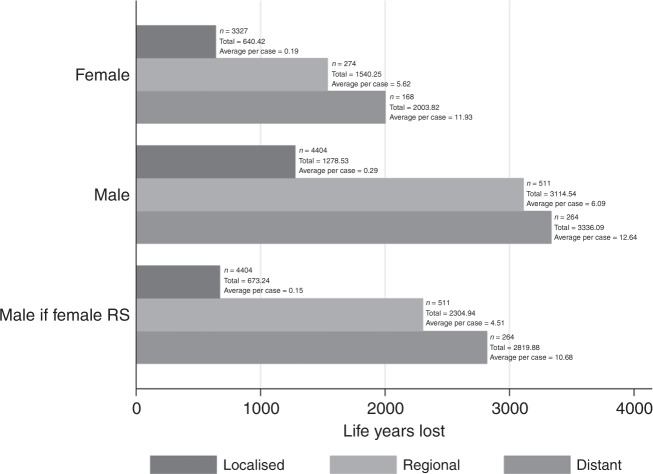
Total life years lost for melanoma skin cancer patients diagnosed in 2017, stratified by sex and stage at diagnosis, and adjustment for potential gain in life years were males to experience the same relative survival as females. Number of cases, total life years lost and average life years lost per case are presented for each stage/sex combination.

Figure 3 shows the TLYL from melanoma cases diagnosed in 2017, as well as the potential gain in life years were males to experience relative survival equal to females. This highlights the disparity in life years being contributed between the sexes, and how sex-specific survival differences impact the overall societal burden of melanoma cancer within a calendar year. Male cases contribute a greater number of life years lost across the population for patients diagnosed in 2017 across all stages at diagnosis, as well as a higher number of cases. In localised cases where the best survival is experienced, males diagnosed in this calendar year contribute 3545 more life years lost when compared to females, as a result of both higher numbers and worse survival. When looking at the stage-specific average life years lost per case, males lose a higher number of life years on average per case in all stage groups. In males in 2017, localised cases account for 85.1% of all melanoma diagnoses, and despite the very small life years lost per case, still are responsible for 16.5% of all life years lost. In females, this changes to 88.3% of all cases contributing 15.3% of life years being lost in the calendar year. In contrast, at the distant stage for males, 5.1% of cases are responsible for 43.2% of life years lost, and in females, 4.5% of cases contribute 47.9% of life years lost.

Across all melanoma cases diagnosed in 2017, a total of 1931.10 life years could be gained if males experienced the same relative survival as females. Across 4404 localised cases, 605.29 life years could be gained, as well as 809.60 life years, and 516.21 life years could be gained from the regional and distant stages from 511 and 264 cases, respectively. Despite having the best stage-specific survival, localised cases comprise a significant volume of potential life years to be gained due to the high proportion in this group. The comparatively small amount of life years to be gained within the distant-stage cases can be attributed to the older-age distribution of patients, as well as the reduction in LEL differences as age at diagnosis progresses within the distant-stage group. Potential improvement at localised stage is responsible for 31.34% of the total potential gain in life years, despite having the smallest per-case impact of the three stages at diagnosis, because of the stage distribution of melanoma cases.

**Fig. 4:**
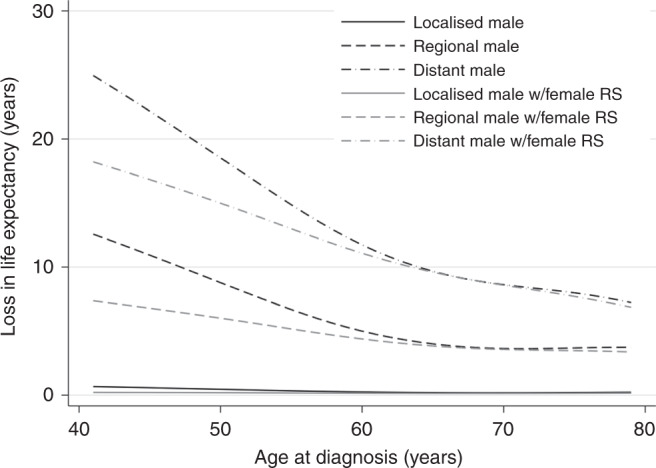
Loss in Expectation of Life if sex differences in survival are eliminated. Stage-specific LEL of males compared with stage-specific LEL of males when assigned female relative survival.

Figure 4 demonstrates the potential reduction in loss-of-life expectancy if sex inequalities in melanoma survival were eliminated. For a 50-year-old male with melanoma, they would gain on average 0.25 years for a localised diagnosis, 2.80 years for a regional diagnosis and 3.43 years for a distant diagnosis were males able to experience the same relative survival as females. The sex differences in LEL vary across age with younger patients subject to more dramatic improvements in survival, as well as variation across stages.

## Discussion

This study investigated the impact of a melanoma skin cancer diagnosis on patients’ life expectancy and the variation within stage and age at diagnosis in the SEER (9 Registries) population. Stage differences were quantified using life-expectancy metrics to assess the lifetime-survival impact following a cancer diagnosis. The sex inequality in melanoma survival in particular was highlighted by estimating the impact of eliminating the relative survival differences and calculating the potential gain in life years by attributing female relative survival to the male population. We find that for diagnoses in a single calendar year, 1931.10 life years could potentially be gained if males experienced the relative survival of females.

The majority of melanoma cases are diagnosed at localised stage and experience very little reduction in life expectancy across the whole range of ages at diagnosis in both male and female cases, in part due to the relative ease of tumour resection where the cancer has not metastasised. Alternatively, at the more advanced stages, there is a considerable reduction in life expectancy compared to the average life expectancy of covariate-matched general population individuals. For example, a 60-year-old male experiences on average a loss in life expectancy of 4.99 years when diagnosed with a regional melanoma case, and this figure rises to 11.73 years for distant diagnoses. The female-equivalent figures were 4.79 and 12.62 years, respectively, highlighting the better prognosis experienced by females. These figures vary strongly by age at diagnosis, with younger patients experiencing the best survival. The majority of melanoma incidence in the United States occurs between 55 and 74, accounting for approximately 45% of all melanoma diagnoses;^27^ however, while the incidence is low for those under the age of 45, it is one of the most common cancers diagnosed among adolescents and young adults,^28^ and it is therefore important to understand the changes in survival trends for the patients diagnosed with melanoma at a younger age.

The variability in loss of expectation of life across sex when estimated across age at diagnosis can be in part attributable to the increased general life expectancy that females experience in comparison to males. Similarly, younger patients have significantly more life years available to lose. Therefore, when comparing loss in life expectancy across sex and age, proportional measures offer an alternative to aid fair comparisons. For example, for subjects diagnosed with regional-stage melanoma at the age of 60, males can expect to lose 4.99 years of life, while females expect to lose 4.79 years. The absolute difference does not account for the varying healthy population life expectancy between the sexes, and so comparing the PELL (25.16 vs. 20.19%) demonstrates a more effective comparison. Similarly, when comparing across ages, males diagnosed with distant- stage melanoma at the age of 80 lose 81.74% of their remaining life, while males diagnosed at distant stage at the age of 50 lose 67.63%, a difference of 14.11%. The use of proportional measures provides easily interpretable and comparable metrics to describe differences in survival across population subgroups while accounting for variation in absolute expected remaining life.

These findings demonstrate patterns in survival within population subgroups that have been suggested previously. There is consistent evidence that stage at diagnosis is a major factor in survival differences within European and American populations,^29,30^ and the disparity between sexes found here is consistent with prior research within Nordic^10^ and the US population,^31^ and reaffirming the influence that age at diagnosis has across the post-diagnosis pathway between sexes.

External factors that have not been accounted for in this analysis may also play a role in driving survival differences in population subgroups. Socioeconomic groups are known to experience differing survival,^32^ and factors such as access to screening and treatment may also influence this. Naturally, some people are more likely to seek care than others and this may be more prevalent in certain subgroups, and adjusting for these known and unknown factors that drive differences in cancer survival is inherently difficult.

Calculating life-expectancy measures derived from flexible parametric survival models allows for metrics that are easily communicated and simple to interpret for non-statisticians. Loss in life expectancy provides a method for assessing post-diagnosis pathways in a variety of covariate- specific individuals, as well as providing a base to quantify the overall societal burden. By also presenting proportional measures, fair comparisons can be made across a variety of population subgroups. Estimating the overall burden on the population is simple, requiring only the survival estimates and the number of cancer cases diagnosed in a given period of time.

With increasing global melanoma incidence and mortality, it is important to identify variations in survival across population subgroups. By doing so, it may become possible to identify the highest- risk groups and drive change to implement better screening and awareness to try and reduce the incidence of melanoma cases, as well as identify cases at earlier stages where survival is significantly better. By improving the stage distribution of melanoma cases, improvement would be seen in survival at both the individual and population level. By eliminating sex differences in survival, many life years could be gained each year within the SEER population; however, further research is required to better understand the biological and behavioural mechanisms that drive these disparities.

## Supplementary information

Statistical Methods Supplement

## Data Availability

All data used in this paper (individual case listings as well as US population mortality data) may be accessed and analysed via the SEER*Stat web program following the submission of a request for access to the data at https://seer.cancer.gov/seertrack/data/request/.

## References

[1] Linos E, Swetter S, Cockburn M, Colditz G, Clarke C (2009). Increasing burden of melanoma in the United States. J. Invest. Dermatol..

[2] Ferlay. J., Colombet. M. & Bray F. Cancer incidence in five continents, CI5*plus:* IARC CancerBase No. 9. https://ci5.iarc.fr (International Agency for Research on Cancer, Lyon, France, 2018).

[3] Guy G, Thomas C, Thompson T, Watson M, Massetti G, Richardson L (2015). Vital signs: melanoma incidence and mortality trends and projections—United States, 1982–2030. Morbidity Mortal. Wkly. Report..

[4] Whiteman D, Green A, Olsen C (2016). The growing burden of invasive melanoma: projections of incidence rates and numbers of new cases in six susceptible populations through 2031. J. Invest. Dermatol..

[5] Singh P, Hee Jin K, Schwartz R (2016). Superficial spreading melanoma: an analysis of 97,702 cases using the SEER database. Melanoma Res..

[6] Matthews, N., Li, W., Qureshi, A., Weinstock M. & Cho E. Epidemiology of melanoma. *Cutaneous Melanoma: Etiology and Therapy*. 10.15586/codon.cutaneousmelanoma.2017.ch1 (2017).

[7] Kronin K, Feuer E (2000). Cumulative cause-specific mortality for cancer patients in the presence of other causes: a crude analogue of relative survival. Stat. Med..

[8] Pohar Perme M, Stare J, Esteve J (2011). On estimation in relative survival. Biometrics.

[9] Cook B, McGlynn K, Devesa S, Freedman N, Anderson W (2011). Sex disparities in cancer mortality and survival. Cancer Epidemiol., Biomark. Prev..

[10] Radkiewicz C, Johansson A, Dickman P, Lambe M, Edgren G (2017). Sex differences in cancer risk and survival: a Swedish cohort study. Eur. J. Cancer.

[11] Dickman P, Coviello E (2015). Estimating and modelling relative survival. Stata J..

[12] Andersson T, Dickman P, Eloranta E, Sjovall A, Lambe M, Lambert P (2015). The loss in expectation of life after colon cancer: a population-based study. BMC Cancer.

[13] Rutherford M, Andersson T, Moller H, Lambert P (2015). Understanding the impact of socioeconomic differences in breast cancer survival in England and Wales: avoidable deaths and potential gain in expectation of life. Cancer Epidemiol..

[14] Bower H, Björkholm M, Dickman P, Hoglund M, Lambert P, Andersson T (2016). The life expectancy of chronic myeloid leukaemia patients is approaching the life expectancy of the general population. J. Clin. Oncol..

[15] Surveillance, Epidemiology, and End Results (SEER) Program (www.seer.cancer.gov) SEER*Stat Database: Mortality—All COD, Aggregated With State, Total U.S. (1969-2017), National Cancer Institute, DCCPS, Surveillance Research Program, released December 2019. Underlying mortality data provided by NCHS (www.cdc.gov/nchs).

[16] Ruhl J, Adamo M, Dickie L (2016). SEER Program Coding and Staging Manual 2016: Section V..

[17] Royston P, Palmer M (2002). Flexible parametric proportional-hazards and proportional-odds models for censored survival data, with application to prognostic modelling and estimation of treatment effects. Stat. Med..

[18] Nelson C, Lambert P, Squire I, Jones D (2007). Flexible parametric models for relative survival, with application in coronary heart disease. Stat. Med..

[19] Lambert P, Royston P (2009). Further development of flexible parametric models for survival analysis. Stata J..

[20] Syriopoulou E, Mozumder S, Rutherford M, Lambert P (2018). Robustness of individual and marginal model-based estimates: A sensitivity analysis of flexible parametric models. Cancer Epidemiol..

[21] Falcaro M, Nur U, Rachet B, Carpenter J (2015). Estimating excess hazard ratios and net survival when covariate data are missing: strategies for multiple imputation. Epidemiology.

[22] Carpenter J, Kenward M (2013). Multiple Imputation and its Application..

[23] Brenner H, Söderman B, Hakulinen T (2002). Use of period analysis for providing more up-to-date estimates of long-term survival rates: empirical evaluation among 370,000 cancer patients in Finland. Int. J. Epidemiol..

[24] Brenner H, Hakulinen T (2007). Maximising the benefits of model-based period analysis of cancer patient survival. Cancer Epidemiol., Biomark. Prev..

[25] Andersson T, Dickman P, Eloranta E, Lambe M, Lambert P (2013). Estimating the loss in life expectation of life due to cancer using flexible parametric models. Stat. Med..

[26] StataCorp (2019). Stata Statistical Software: Release 16..

[27] Surveillance, Epidemiology, and End Results (SEER) Program (www.seer.cancer.gov) SEER*Stat Database: Incidence - SEER Research Data, 9 Registries, Nov 2019 Sub (1975-2017)—Linked To County Attributes—Time Dependent (1990–2017) Income/Rurality, 1969–2017 Counties, National Cancer Institute, DCCPS, Surveillance Research Program, released April 2020, based on the November 2019 submission.

[28] Watson M, Geller A, Tucker M, Guy G, Weinstock M (2016). Melanoma burden and recent trends among non-Hispanic whites aged 15–49 years, United States. Prev. Med..

[29] Svedman F, Pillas D, Taylor A, Kaur M, Linder R, Hansson J (2016). Stage-specific survival and recurrence in patients with cutaneous malignant melanoma in Europe—a systematic review of the literature. Clin. Epidemiol..

[30] Enninga E, Moser J, Weaver A, Markovic S, Brewer J, Leontovich A (2017). Survival of cutaneous melanoma based on sex, age, and stage in the United States, 1992-2011. Cancer Med..

[31] Khosrotehrani K, Dasgupta P, Byrom L, Youlden D, Baade P, Green A (2015). Melanoma survival is superior in females across all tumour stages but is influenced by age. Arch. Dermatological Res..

[32] Syriopoulou E, Bower H, Andersson T, Lambert P, Rutherford M (2017). Estimating the impact of a cancer diagnosis on life expectancy by socio-economic group for a range of cancer types in England. Br. J. Cancer.

