# Supplementary material for: Understanding the impact of sex and stage differences on melanoma cancer patient survival: a SEER-based study
Source: Br J Cancer. 2020 Nov 4;124(3):671–7. doi: 10.1038/s41416-020-01144-5 (PMC7851379; doi:10.1038/s41416-020-01144-5)
Supplement: Supplementary file 1 — Statistical Methods Supplement [file 41416_2020_1144_MOESM1_ESM.docx]

Supplementary Table 1: Stage Missingness Distributions across Imputation Model Variables

|  | Stage at Diagnosis | | | |  |
| --- | --- | --- | --- | --- | --- |
|  | Localised | Regional | Distant | Missing | Total |
| Age Group | | | | | |
| Under 45 | 15 775  (76.55%) | 1 805  (8.76%) | 404  (1.96%) | 2 623  (12.73%) | 20 607 |
| 45-60 | 23 051  (79.71%) | 2 363  (8.17%) | 914  (3.16%) | 2 592  (8.96%) | 28 920 |
| 60-75 | 26 992  (79.46%) | 3 065  (9.02%) | 1 279  (3.77%) | 2 633  (7.75%) | 33 969 |
| Over 75 | 15 924  (73.27%) | 2 491  (11.62%) | 957  (4.46%) | 2 070  (9.65%) | 21 442 |
| Total | 81 742  (77.90%) | 9 724  (9.27%) | 3 554  (3.39%) | 9 918  (9.45%) | 104 938 |
| Primary Tumour Sub-site | | | | | |
| C44.0 | 157  (62.30%) | 49  (19.44%) | 9  (3.57%) | 37  (14.68%) | 252 |
| C44.1 | 565  (66.63%) | 83  (9.79%) | 34  (4.02%) | 166  (19.58%) | 848 |
| C44.2 | 2 497  (85.02%) | 243  (8.27%) | 43  (1.46%) | 154  (5.24%) | 2 937 |
| C44.3 | 8 185  (80.47%) | 923  (9.07%) | 157  (1.54%) | 907  (8.92%) | 10 172 |
| C44.4 | 6 355  (74.33%) | 1 208  (14.13%) | 234  (2.74%) | 753  (8.81%) | 8 550 |
| C44.5 | 27 284  (84.17%) | 2 698  (8.32%) | 530  (1.63%) | 1 905  (5.88%) | 32 417 |
| C44.6 | 21 228  (86.48%) | 1 814  (7.39%) | 286  (1.17%) | 1 219  (4.97%) | 24 547 |
| C44.7 | 14 928  (77.70%) | 2 031  (10.57%) | 316  (1.64%) | 1 938  (10.09%) | 19 213 |
| C44.8 | 62  (28.84%) | 13  (6.05%) | 6  (2.79%) | 134  (62.33%) | 215 |
| C44.9 | 481  (8.31%) | 662  (11.44%) | 1 939  (33.51%) | 2 705  (46.74%) | 5 787 |
| Total | 81 742  (77.90%) | 9 724  (9.27%) | 3 554  (3.39%) | 9 918  (9.45%) | 104 938 |
| Tumour Grade | | | | | |
| Grade 1 | 310  (76.54%) | 60  (14.81%) | 5  (1.23%) | 30  (7.41%) | 405 |
| Grade 2 | 454  (74.67%) | 94  (15.46%) | 14  (2.30%) | 46  (7.57%) | 608 |
| Grade 3 | 442  (58.08%) | 194  (25.49%) | 84  (11.04%) | 41  (5.39%) | 761 |
| Grade 4 | 187  (52.82%) | 107  (30.23%) | 36  (10.17%) | 24  (6.78%) | 354 |
| T-Cell | 571  (18.19%) | 92  (2.93%) | 61  (1.94%) | 2 415  (76.94%) | 3 139 |
| B-Cell | 281  (14.29%) | 19  (0.97%) | 51  (2.59%) | 1 616  (82.16%) | 1 967 |
| Null-Cell | 0  (0%) | 0  (0%) | 0  (0%) | 3  (100%) | 3 |
| NK Cell | 0  (0%) | 1  (5.88%) | 1  (5.88%) | 15  (88.24%) | 17 |
| n/a | 79 497  (81.38%) | 9 157  (9.37%) | 3 302  (3.38%) | 5 728  (5.86%) | 97 684 |
| Total | 81 742  (77.90%) | 9 724  (9.27%) | 3 554  (3.39%) | 9 918  (9.45%) | 104 938 |

**Statistical Methods Appendix**

Flexible parametric models were fitted with restricted cubic splines with 5 degrees of freedom to better capture the shape of the baseline excess hazard. Restricted cubic splines are flexible mathematical functions defined by piecewise polynomials which are forced to be linear before the first and after the last knot. Knots were located at the 25^th^, 50^th^, 75^th^ and 95^th^ centile position. Time varying covariates were implemented using the stage and age variables, with 3 degrees of freedom and knots placed at the 33^rd^ and 67^th^ centile position. Age and stage at diagnosis were included in the model as time varying covariates based on likelihood ratio tests to test model fit. An age and stage interaction term was considered but was found not to improve the model and hence was not included.

Age was incorporated into the model using splines to allow for non-linear effects as a continuous variable. Survival estimates were Winsorized based on age at diagnosis in order to limit the influence of extreme age values below the 2^nd^ and above the 98^th^ percentile of the age distribution.

To estimate relative survival metrics, it is necessary to incorporate life table estimates which correspond to the population under analysis. Here, SEER provide their own population mortality figures which align with the database of patients in terms of age, sex and race, and cover specifically the US population.

Multiple imputation was conducted to generate data for missing stage at diagnosis information. This process was performed using multinomial logistic regression incorporating the Nelson-Aalen estimate of the all-cause cumulative hazard and the event indicator. Rubin’s rules were implemented in order to accurately construct combined point estimates and confidence intervals of survival estimates across the imputed datasets.

Loss in expectation of life was estimated across the range of stages at diagnosis as well as across a full range of ages. Loss in expectation of life was estimated on the absolute and proportional scale to provide a foundation for better comparison between stages, ages and sexes. Loss in expectation of life is defined as the difference between the life expectancy in a cancer-free populations and that of a cohort of cancer patients matched by age, sex and year of diagnosis. The general population data in the form of a population mortality table acts as a proxy for the cancer-free population. Life expectancy is calculated as the area under the survival curve, and LEL can be written as: $LEL\left( x \right)=\int_{0}^{t_{max}} S^{*}\left( t,x^{'} \right)dt- \int_{0}^{t_{max}} S\left( t,x \right)dt.$

The remaining life expectancy metrics can be presented as a function of LEL. Proportion of Expected Life Lost can be written as $PELL=\frac{LEL}{Expected Remaining Life Years}$, total life years lost can be written as $TLYL=N\cdot LEL$ , and potential gain in life years can be written as $PGLY=N\cdot\left( {LEL}_{alt}-LEL \right),$where ${LEL}_{alt}$ represent the loss in life expectancy of an alternative patient population. In this instance, ${LEL}_{alt}$ represents males who were assigned the relative survival of females.
